# Integration at the Round Table: *Marine Spatial Planning in Multi-Stakeholder Settings*

**DOI:** 10.1371/journal.pone.0109964

**Published:** 2014-10-09

**Authors:** Erik Olsen, David Fluharty, Alf Håkon Hoel, Kristian Hostens, Frank Maes, Ellen Pecceu

**Affiliations:** 1 Institute of Marine Research, Bergen, Norway; 2 School of Marine and Environmental Affairs, University of Washington, Seattle, Washington, United States of America; 3 Institute of Marine Research, Tromsø, Norway; 4 Animal Sciences, Aquatic Environment and Quality, Bio-environmental Research, Institute for Agricultural and Fisheries Research (ILVO), Oostende, Belgium; 5 Maritime Institute, Ghent University, Ghent, Belgium; University of Waikato (National Institute of Water and Atmospheric Research), New Zealand

## Abstract

Marine spatial planning (MSP) is often considered as a pragmatic approach to implement an ecosystem based management in order to manage marine space in a sustainable way. This requires the involvement of multiple actors and stakeholders at various governmental and societal levels. Several factors affect how well the integrated management of marine waters will be achieved, such as different governance settings (division of power between central and local governments), economic activities (and related priorities), external drivers, spatial scales, incentives and objectives, varying approaches to legislation and political will. We compared MSP in Belgium, Norway and the US to illustrate how the integration of stakeholders and governmental levels differs among these countries along the factors mentioned above. Horizontal integration (between sectors) is successful in all three countries, achieved through the use of neutral ‘round-table’ meeting places for all actors. Vertical integration between government levels varies, with Belgium and Norway having achieved full integration while the US lacks integration of the legislature due to sharp disagreements among stakeholders and unsuccessful partisan leadership. Success factors include political will and leadership, process transparency and stakeholder participation, and should be considered in all MSP development processes.

## Introduction

The management of marine ecosystems and their human activities underwent dramatic changes since the 1992 Rio Declaration on Environment and Development. Today sustainable development is still a fundamental principle, but following the 2002 Johannesburg Declaration, sustainable management has been expanded to encompass not only single species or sectors, but the whole ecosystems through the “ecosystem approach to management” (EBM) [1], [2]. EBM is a powerful concept based on analysing and managing the ecosystems from a holistic approach, taking into account all components, pressures and impacts [3], [4].

Because ecosystems are spatially explicit, area-based management approaches offer a suitable and efficient way of implementing EBM into practice [5], [6]. One such approach is marine spatial planning (MSP), which offers an effective perspective to deal with the challenging issues of multiple use and multiple (cumulative) impacts in EBM [7]. Even though a spatial perspective has been successful in terms of leading to novel and forward-looking ecosystem-based management plans, like in Norway and Australia [8]–[11], the development of marine spatial plans is a complex process at the borders between science, management and politics. Several approaches, like the UNESCO 10-step approach [12], have been developed to lead the practitioners safely from start to finish during planning. The establishment of effective governance of the planning, implementation and review processes, is a fundamental step to develop sound MSP [12], [13].

Because MSP by definition is multi-sectoral, a potentially large number of managers, stakeholders and policy-makers are involved, each accustomed to operate on his own (*i.e.* within specific sectors). Successful MSP means getting all these actors to communicate and work together in an integrated way. Therefore, integration means crossing boundaries at professional, physical, institutional or administrative level [14]. To develop the appropriate measures in an integrated (ecosystem) MSP setting, the integration of concerns and interests across sectors (horizontal integration) and between governmental levels or between government and stakeholders (vertical integration) is required. Integration is fundamental to MSP and especially important to pro-actively resolve spatial conflicts [14].

**Figure 1:**
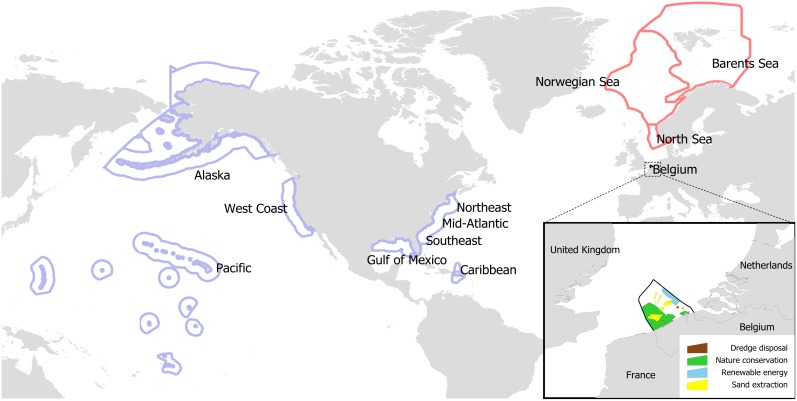
Marine spatial planning (MSP) study areas around the North Atlantic. United States (blue), Norway (red) and Belgium (Green, detail in inset). The US ocean policy area is divided into 9 planning areas (named in figure) that span the EEZ, but the borders between each of these areas have not been officially defined.

In this paper we explore how such integration has been dealt with in three markedly different MSP processes: the Belgian MSP covering the whole Belgian EEZ in the North Sea, the Norwegian Integrated Management plans – three plans covering the Norwegian EEZ, and the current US National Ocean Policy (Figure 1) regional planning process now being implemented. We illuminate how the integration of concerns and interests varies with the context and commonalities that allow for a successful integration of different viewpoints and hence successful governance of MSP.

## The Three Case Studies

### Belgium: from Masterplan to MSP

In March 2014, Belgium approved a legally binding Marine Spatial Plan (Royal Decree of 20 March 2014 on the adoption of the MSP) for the Belgian part of the North Sea (BPNS), a small area of ca. 3500 km^2^ covering the territorial sea, the continental shelf and the entire Belgian Exclusive Economic Zone (EEZ). MSP in Belgium is the result of a long process, going back to initial attempts to implement the 1999 Marine Environmental Protection Act (MEPA), followed by the so-called “Masterplan for the BPNS” in 2003–2005. Since 2012 an explicit legal basis for MSP in Belgium is embedded in the MEPA, which clearly indicates the environmental roots of MSP. Therefore, governments and users of the sea in Belgium have to take into account several legal principles, namely the principle of prevention, the precautionary principle, the polluter pays principle and the restoration principle (art. 5 MEPA).

**Figure 2:**
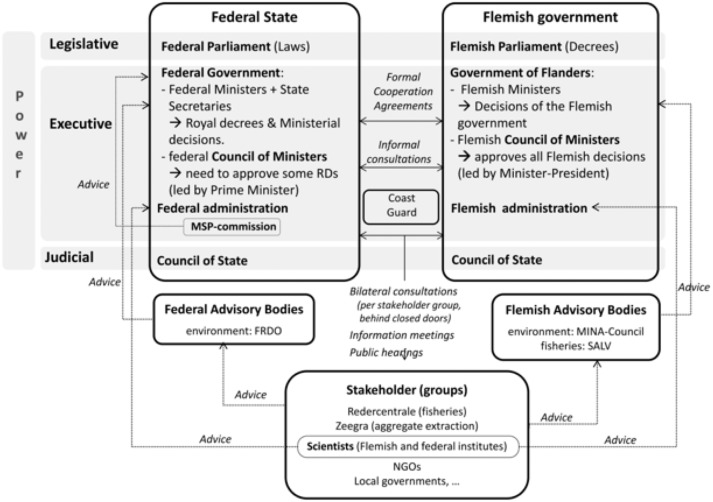
Institutional and stakeholder integration in Belgium. Institutional bodies, stakeholder participation and integration, related to marine and maritime governance in the Belgian part of the North Sea.

Belgium functions as a multi-level government with authorities divided among local, regional, federal (and European) levels. The marine competences are divided between the federal state and the Flemish region, and within each level, competences are fragmented over several departments [15]. No less than 17 governmental institutions (both Flemish and federal) have some form of competence at sea. Both governments are legally equal in adopting their legislation within their fields of competence (Figure 2). Consultation between the two governments can be held at ministerial level, between their cabinets or between their administrations. Matters in which both governments have different exclusive competences can be jointly dealt with in formal cooperation agreements.

It took until 2003, with the appointment of a federal Minister for the North Sea, to set a major step forward in the Belgian MSP process. This Minister was mandated to spatially co-ordinate all activities and competences at sea, except for fisheries which is a regional (Flemish) competence. He initiated a more strategic approach to the (potential) conflicting claims between users of the BPNS which emerged between 1999 and 2003. He developed the BPNS Masterplan, which actually combined several political decisions concerning marine matters that were taken by the federal Council of Ministers and implemented by a number of Royal decrees. The plan was further adopted in two phases: in 2003 with an agreement on the delimitation of several zones for two economic activities- aggregate extraction and offshore renewable energy - and later in 2005 with the delimitation of nature conservation areas for birds and habitat protection. However, it remained essentially a zoning plan, without a legal basis for a planning process or an integrated policy approach, nor a clear and transparent process for stakeholder involvement and public participation [16].

In Belgium, there is strong vertical integration between the highest levels (Ministers) and their public administrations. Moreover, each governmental level has its own advisory bodies (Figure 2), representing the major stakeholders, e.g. the Federal Council for Sustainable Development (FRDO), the Flemish Council for Environment and Nature (MiNa Council) and the Flemish Strategic Advisory body for Agriculture and Fisheries (SALV). It is important to recognize that despite the existence of sectoral cooperation agreements and advisory bodies, the concerned parties only have the mandate and responsibility for their own competences.

Stakeholders have been involved in the MSP process since the Masterplan was presented in 2003, but this happened only on a sectoral basis and mostly through bilateral consultations behind closed doors [17]. This was in line with an old Belgium practice of informal consultations within and between the governments and administrations, as well as between the governments and stakeholders. As such the Masterplan was drawn up on an ‘*ad hoc*’ basis, taking into account the individual and selective demands of sectoral organizations and individual stakeholders. As a consequence, consultation with other sea users than the ones in the focus of the Masterplan was not deemed necessary. After 2006, the Masterplan has been adjusted a few times, in particular to take into account shipping interests and to designate a new marine protected area (2008–2010). However, these developments were once again dealt with at sectoral level.

In 2012, the newly elected federal government re-appointed the same Minister for the North Sea, and a second phase of MSP started. The new marine spatial plan departed from the BPNS Masterplan, but with a clear aim to establish a ‘legally binding’ marine spatial plan and process. The draft spatial plan, which was again based on informal and formal consultations with stakeholders, was subjected to a series of administrative consultations and advice from a new Advisory Commission on MSP. The latter was formally established by a Royal Decree in November 2012 and involves all federal authorities with competences at sea, next to ‘invited’ Flemish authorities. As such, an integration between all federal governmental departments concerning MSP became formally embedded in the new MSP legislation.

Next to the MSP Advisory Commission, experts were consulted, stakeholder participation was organized and a strategic environmental assessment (SEA) of the spatial plan was conducted. In July 2013, an updated plan was approved by the Council of Ministers, followed by consultation with the public and neighboring countries and advice from advisory bodies and regional authorities. The Ministry of Environment took into account 140 remarks and finally the Council of Ministers approved the MSP by Royal Decree on 20 March 2014.

The new Belgian marine spatial plan comprises the coordinates of all delimitated zones for the activities that are allowed and limited or prohibited in the BPNS, including some new zones for future activities (energy atolls, sustainable aquaculture, etc.). The Royal Decree also contains several annexes outlining the spatial context and explaining the policy choices that have been made. The long term vision to implement MSP in Belgium foresees concrete objectives for the period 2014–2020 and a revision of the spatial plan every six years, although in between adaptation of the plan is not excluded.

A major shortcoming of the Belgian MSP might be that fisheries is only partly included in the MSP, as this is a not a competence of the federal government (Flemish competence). Nevertheless, consultations took place at the highest political (ministerial) level, and certain limitations for fisheries are included in the new MSP, such as the prohibition of fisheries in the offshore renewable energy concession zones, and a limitation for certain fishing techniques (mainly classic bottom disturbing trawling techniques) in specific parts of the Natura 2000 area ‘Vlaamse Banken’ in order to achieve a good environmental status for the BPNS.

### Norway: Integrated Management Plans

As a response to the international [1] and regional drivers (North Sea ministerial conference in 1997) for EBM as well as national pressures from the petroleum industry to get access to new areas further north and along the coast, the new government heralded marine Integrated Management Plans (IMPs) in its coming-to-power declaration in 2001 [18]. The development of these IMPs started in 2002, with the first plan, for the Lofoten–Barents Sea area implemented in 2006 [8], [18], the Norwegian Sea plan in 2009 [9], and the last plan for the North Sea and Skagerrak area in 2013 [19]. These IMPs are regional plans integrating all human uses of the area in a spatial context through sectoral zoning and geographical analysis. The Lofoten-Barents Sea plan has been revised once in 2011, the Norwegian Sea plan is due for revision in 2014.

An inter-ministerial steering group led by the Ministry of Environment was set up to coordinate the development and implementation of these IMPs. The steering group had members from all relevant sector-ministries (e.g., Foreign Affairs, Fisheries and Coastal Affairs, Petroleum and Energy). This strong political (top-down) steering group tasked the different institutions and directorates under each ministry to contribute to the development and implementation of the plan. Each plan was customized for the particular ecosystem, but with a similar overarching strategic objective: “*to allow for sustainable use while ensuring continued ecosystem health*” [20]. The duality in this main strategic objective reflects the underlying conflict to integrate two widely different objectives, namely the push for increased industrial (petroleum) developments and the protection of the ecosystems (to ensure the health and survival of species and habitats) [21].

**Figure 3:**
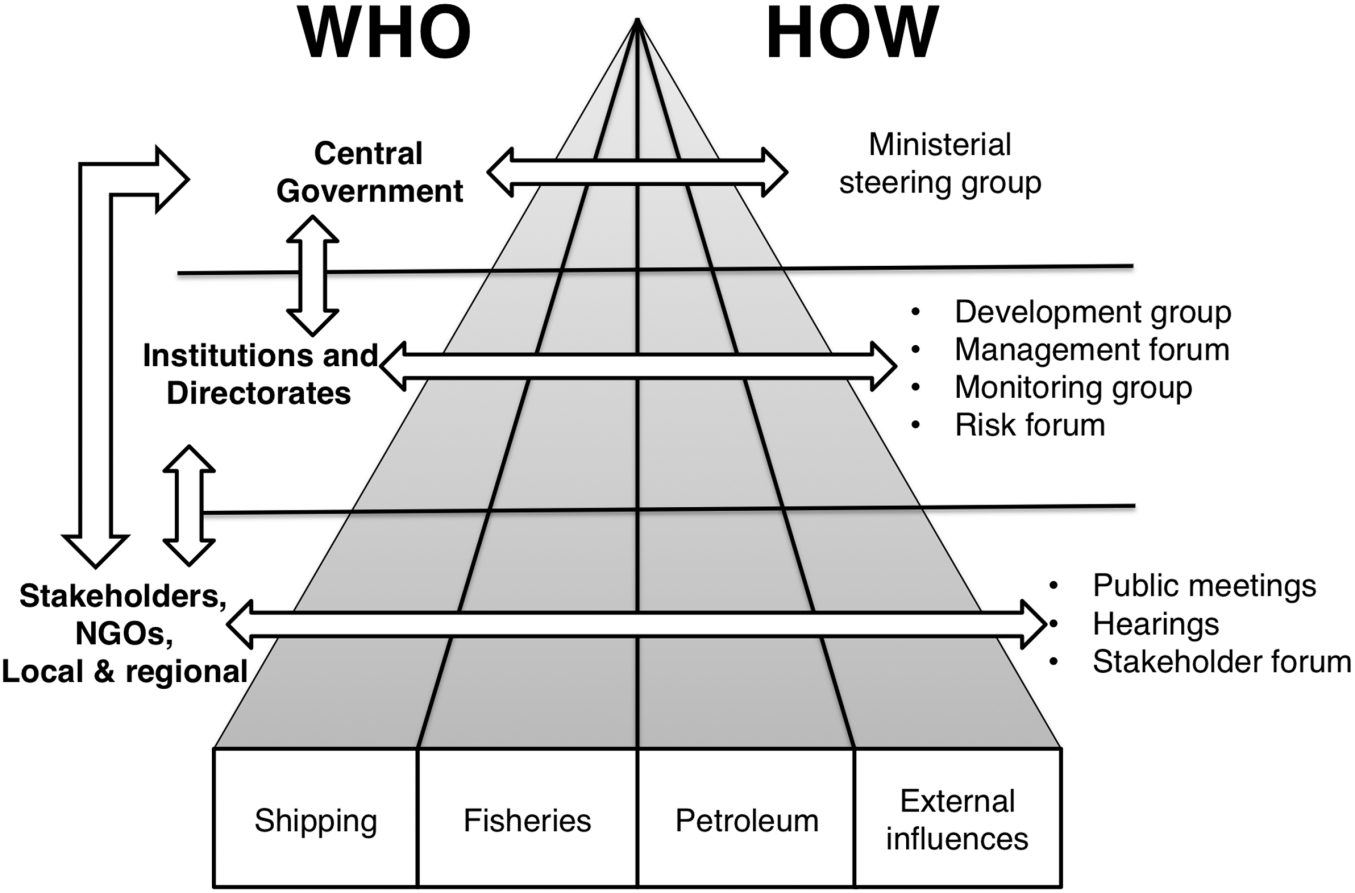
Institutional, government, and stakeholder integration in Norway. Institutional bodies, stakeholder participation and integration, related the development and implementation of the Integrated Management plan for the Lofoten – Barents sea area.

Integration of different interests and concerns is central to the Norwegian MSP process, and already commenced at the start of the planning process in 2002. The various sectors and levels (of government) have been integrated by jointly developing the knowledge base and management measures across sectors and between levels of government (Figure 3). Joint groups, forums and meeting places across sectors have been set up to achieve horizontal – cross-sectoral integration. Hearings, open public meetings and sectoral meetings with the government help to foster cooperation between the various governmental levels and with stakeholders in particular.

The Norwegian planning process is centralized, where decisions are made by the government and subjected to parliamentary approval. As such, all IMPs have been passed with broad political approval in the Parliament. Involvement of local government levels (e.g. counties) has been poor, mainly because ocean management is a national issue in Norway, with only limited local control over marine resources or management of marine space.

The overarching conflict of industrial use versus nature protection has been a central issue for debate at all levels, in particular in the management and risk forums (Figure 3). In these multi-sector groups the different views allowed for cross-sectors insight and discussion, and although this has not resolved the conflict, it has increased mutual understanding and respect, based on the personal, professional and institutional relationships that have been established during the planning and implementation process. The networking has increased trust among the participants, which made it possible to discuss difficult and challenging issues.

Imposing consensus between the sectoral government institutions was instrumental to develop and implement the Lofoten-Barents Sea plan in a 4-year time frame. This first IMP spearheaded the development of the other plans and created much of the structure and methods to allow the two later plans to be created in 3-year processes. However, seeking consensus could also lead to suppression of substantial and value-based differences. It can be questioned whether the decision-makers would be better served if the differing views and options in the central conflict (industrial use vs. nature conservation) would have been clearly presented as different choices rather than hidden in a compromise. A consensus approach limits the options for the decision-makers, who normally prefer a range of options which allows them more room to govern.

The existing sectoral legislation and management structures have been the main barrier for integration. Using soft-law in processes like the development of IMP’s, is a tradition in Norway, and is fast, pragmatic and efficient. Also, by implementing through existing legislation and institutional structures, the policy is rapidly and effectively translated into practice without spending much time on developing new legislation or governance structures. However, this approach does little to integrate concerns across the existing sectoral legislation. Nevertheless, many new sectoral laws passed since 2002, took the integrated management approach into consideration and specifically required integration between sectors.

### US: Ocean Policy Executive Order

**Figure 4:**
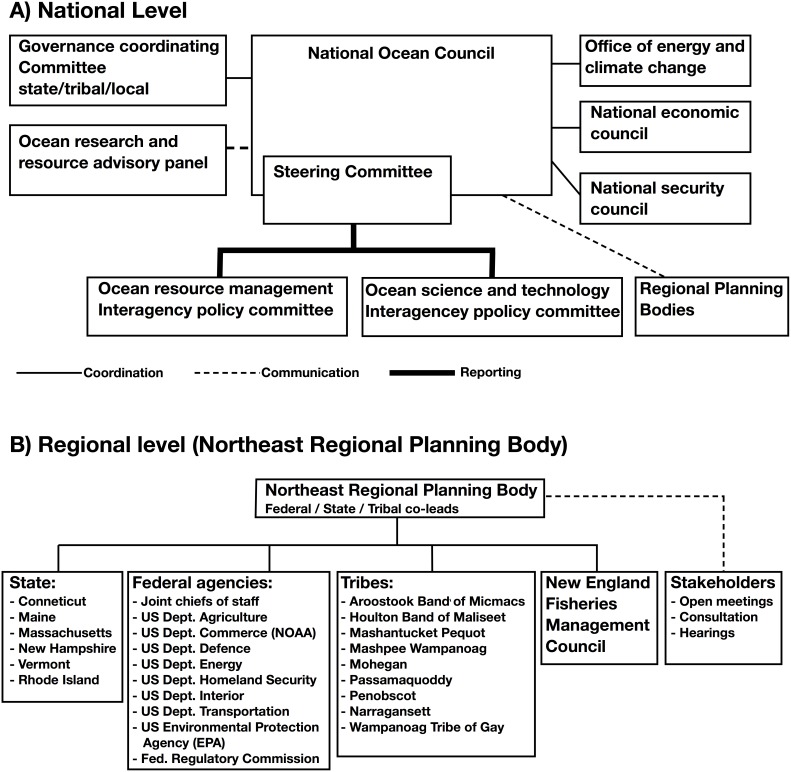
Governance structure and integration in the US Ocean Policy development. A) At a national level leading the Ocean Policy development. B) Regional implementation in regional planning bodies (RPB), exemplified by the Northeast RPB.

Influenced by international MSP developments and strongly promoted by major environmental organizations, the US started a process towards developing an Ocean Policy [22] including MSP. The first steps were already taken by the Bush administration, but development efforts increased in 2008 with the new Obama administration. After 18 months of intensive planning, the National Ocean Policy was effectuated through President Obama’s Executive Order 13547 (July 19, 2010). This Executive Order established the National Ocean Council, consisting of 27 federal agencies, departments and offices. Additionally, the US marine and Great Lakes areas are divided into nine regions, which can form regional planning bodies on a voluntary basis to develop the planning process in their respective regions (Figure 4). The National Ocean Council published an Implementation Plan [23], that further gives guidance to the administration and partner institutions on how the Ocean Policy should be implemented [24]. The Obama Ocean Policy approach relies on existing legal mechanisms [25].

The Ocean Policy can be seen as a MSP process, although the term ‘marine spatial planning’ is not used in the most recent documents as it has become politicized to the point that the US Congress in 2012 and 2013 refused to fund any MSP related activities. The majority in the House of Representatives see MSP as an unnecessary layer of government, hampering the development of business activities at sea. Also industrial (oil, fishermen, etc.) lobby groups (e.g., National Ocean Policy Coalition, see http://oceanpolicy.com) advocated against MSP. Therefore, MSP implementation has ended up in the middle of the partisan battlefield, even though the first steps were taken by the Bush Administration with bipartisan support. As a result, the federal support for the Ocean Policy has decreased compared to what was anticipated in 2010. Currently, the Ocean Policy implementation is totally dependent on support from the State and regional levels, who are leading the way while the federal level lags behind. Substantial financial support has however been provided by private donors to MSP efforts in the Northeast and Mid-Atlantic regions. It is critically important that top-down and bottom-up processes are linked in the implementation of the US Ocean Policy [26], however the situation today is charitably defined as experimental and otherwise defined as chaotic when looking across all nine regions.

Some States have taken a leadership positions in MSP for their coastal environments, e.g., Rhode Island, Massachusetts, Oregon, New York, Hawaii and Florida, with Washington State not far behind [27]. Also, California has taken major steps to develop marine spatial planning through a marine reserve network in state waters. In addition, Maine, New Hampshire, Massachusetts, Rhode Island and Connecticut together with federal partners formed the Northeast Regional Planning body (RPB) as envisioned by the Ocean Policy (Figure 4). The Northeast RPB constituted itself, meets regularly, and is on its way to develop a regional plan in 2015. Also, the Mid-Atlantic and Southeast Atlantic have formed RPBs, with the Mid-Atlantic having started a planning process in 2014, while the Southeast is a bit delayed by the limited participation from Florida. These RPBs are expected to produce regional MSPs by 2015 for review and approval by the National Ocean Council but this timeline is ambitious.

Other regions, except Alaska, have taken up on the Ocean Policy and have or are in the process of forming RPBs. Hawaii (part of the Pacific region) had a state plan under development when the Ocean Policy was being put forward. The greatest challenge in the Pacific region is distance, as it includes Hawaii and all US Pacific Islands, spanning seven time-zones and the international date-line. Finding time, place and funding to bring regional representatives together, is logistically very demanding. The Pacific RPB may ultimately adopt a more archipelagic approach as has been done for fisheries management [28].

The National Ocean Policy envisions a regional approach to develop MSPs for large management regions, *in casu* ecosystems, based on the regional administrative capacity. However, it is recognized that the desire for standard approaches across the nation may not be accommodated at the regional level. Therefore, a flexible structure of Federal agency engagement with States and local governments is required. All current RPBs have wide participation of federal, state and tribal governmental institutions that have formal mandates for ocean management (Figure 4). The RPB work and meetings are open to the public and public interaction is actively encouraged through specific sessions where the public is allowed to ask questions or make comments to the full RPB. These occasions are actively used by self-selected and motivated NGOs, interest groups and the public to voice their perspectives.

## Discussion - Comparing Integration in the Three Cases

### Spatial scale of the managed area

Belgium, Norway and the US represent three different approaches of implementing MSP, stemming from both geography and governance [26]. Whereas the Belgium plan spans the whole EEZ and Belgian national waters, an area of a few thousand square kilometres, both the Norwegian and US plans span millions of square kilometres under different climate and biogeographic regions, making them much more complex than the Belgium plan area. To better handle the varied climatological and geographic patterns, both the US and Norway chose to divide their marine areas into (eco)regions and are developing separate plans for each area. Regardless of the size of the spatially managed area, it remains a complex issue to manage all types of human activities under one umbrella, requiring integration between different sectors, stakeholders and governmental levels. Reducing the size of the planning area will reduce complexities, but only to a certain level, as the multi-sectoral cross-political aspects remain, irrespective of spatial scale.

### Legislation, political will and leadership

The three countries differ markedly in terms of governance: Belgium is an EU member and marine governance has to adhere to EU policies (e.g. Common Fisheries Policy) and directives (e.g., Habitats and Bird, Marine Strategy Framework, Water Framework). Belgium has a strong formal division of power between the federal and regional levels, although integration between both governmental levels is assured through formal agreements and informal consultations. This is also reflected in the protracted Belgian MSP process: after more than 10 years, the MSP plan and process are firmly embedded in environmentally driven legislation.

Norway is not a member of the EU and has an ethnically homogenous population. The government is well integrated across sectors both at central and regional levels. The Norwegian Integrated Management Plans (IMPs) were passed through the Parliament, but are not embedded in Norwegian legislation. They are rather government reports with MSP objectives and a vision on how they will achieve these objectives through a range of management options, including spatial zoning.

In the US the independency of the states led to a geographic division in marine management at the border between federal and state waters, mostly three nautical miles offshore. The US Ocean Policy process, based on a Presidential Executive Order, does not have backing in the Congress, which led to funding restrictions to develop or continue the MSP process. The US Ocean Policy became heavily politicized in 2010 and has since been drawn into a struggle among stakeholders largely along commercial users of ocean space and biodiversity conservation, spilling over into the partisan debates in Congress. Even though embedding MSP in legislation creates a more stable solution in heterogeneous societies like Belgium and the US, achieving overall trust and buy-in is instrumental for a successful implementation (and funding) of the MSP process and plan.

In contrast to Norway and Belgium, broad political support for implementation of MSP has not been reached in the US, due to the lack of strong stakeholder support for MSP by commercial and non-commercial users. Strong political leadership was instrumental to achieve that bipartisan support for MSP in both Belgium and Norway. In Belgium the personal charisma, tenacity and clout of the Minister of the North Sea was pivotal to lead the planning process and persuade the different parties of the necessity of MSP. In Norway it was the tenacity and clout of the Ministry of Environment, strongly supported by the Ministries of Fisheries and Foreign Affairs that pushed the MSP process. Moreover, achieving broad political buy-in was relatively easy in Norway, as the MSP process was partly driven by Norway’s foreign policy. Foreign priorities are very stable in Norway and do not change with changing governments. In the US leadership has certainly been shown at the Executive level but it is not sufficient to carry through to the legislative leadership.

### Drivers and incentives

Leaving aside political and ethnic differences between the countries, socio-economics, i.e. the importance of certain marine industries in the national economy of each country, help to explain some of the differences in their governance approach to MSP. In Belgium, maritime infrastructure (shipping and ports) is very important, next to aggregate extraction and to a lesser extent fisheries. Since 2003 offshore renewable energy (wind-farms) developments have been the main driver for MSP in Belgium. However, adherence to EU environmental directives (Habitats, Bird, Marine Strategy Framework Directive) has created a second driver in MSP in order to identify areas of high biological value (Natura2000 sites) and to achieve a “good environmental status” of the ecosystem. In Norway, the marine natural resources (oil and fish) form the basis for the country’s welfare, and sea-based industries (oil/gas, fisheries and aquaculture) are of fundamental socio-economic importance. It is a high priority for all governmental levels to create (sustainable) marine value in Norway. The health of the marine ecosystem is understood as an important prerequisite for sustainable value creation, but this is not seen as an absolute hindrance to human uses. Therefore, protecting areas by excluding economic activities is not common in Norway.

In the US, sea-based industries, shipping and fishing are of great socio-economic importance, but the country is not as dependent on the exploitation of marine resources (oil/gas and fish) as Norway. The importance and political power of the different sectors vary regionally, e.g., in the Gulf of Mexico the petroleum industry is given priority, while in the Northeast region offshore renewable wind energy has been a driver for MSP. The US has a strong terrestrial tradition of using protection (national parks and forests) as a management measure. This has been transferred to the marine environment with the establishment of several MPAs with a high protection level like the Papahānaumokuākea Marine National Monument (MNM) in the north-western Hawaiian islands, the Marianas Trench MNM and the Pacific Remote Islands MNM.

The economic incentives have led to different objectives in the respective MSP processes. Although all three countries try to combine ecosystem health with economic use, they differ in their approach. While Norway has a clearly stated dual goal of sustainable use while ensuring a healthy ecosystem, Belgium is focusing on protection of valuable habitats and ecosystem components while finding room for new uses and minimizing conflicts. The US objectives are complex and not prioritized in terms of trade-offs between economic use and nature conservation.

### Stakeholder participation and transparency

Integrating stakeholders in the planning process has been important, but done in different ways in the three countries. In Norway and Belgium, stakeholders were brought early on board, which led to acceptance of the MSP process, although with much debate about specific details of the plan. In the US, stakeholders were invited to comment during the 18 month process leading up to the federal Ocean Policy Executive Order. This process built on many years of responding to the US Commission on Ocean Policy and the non-governmental Pew Oceans Commission processes. However, there was considerable surprise that MSP was the most tangible action required by the Executive Order. This shift in approach resulted in several industrial stakeholder groups forming lobby-groups against MSP implementation, which blocked necessary incremental funding of the MSP process. The resultant stakeholder skepticism conveniently fed into the partisan political battle in Washington D.C. In the meantime, stakeholder involvement and transparency is much more effective at the state and regional level in the US. Threats and opportunities are tackled depending on one’s perspective from existing spatial conflicts, and through new drivers primarily associated with both non-renewable (import/export) and renewable (offshore) energy. A credible threat to established interest adds to the acceptance and continuation of the MSP process in most US regions. Full transparency, open meetings, public documents and active solicitation of stakeholder opinions, make the US stand out compared to Belgium and Norway, where transparency was more limited and controlled.

## Conclusion – Integration at the MSP Round Table

Ecosystem-based management in general and MSP in particular is a multifaceted management approach that combines the management of multiple sectors and goals under one roof through integration. Integration can be measured both in relation to geographic scale and governance scope [14] according to which all three cases (Belgian, Norwegian and the US) can be classified as “highly integrated”. However, there are some marked differences between the cases in their horizontal and vertical integration (Table 1).

**Table 1:** Horizontal, vertical and stakeholder integration in the MSP processes in Belgium, Norway and the US.

	Horizontal integration	Vertical integration	Stakeholder integration
**Belgium**	Good integration. Minister for the NorthSea ensured a high government focusand top-level integration. Important federaland partly regional integration achieved	All levels of governmentwell integrated	Consultation and formal rolesin planning group
**Norway**	Good integration both at top governmentlevels and at government institutional levels	All levels of governmentintegrated	Consultation and participationin some meetings
**US**	Good integration between sectors at stateand regional levels where direct threats oropportunities are perceived. Poor integrationat national level due to lack of prioritizationof objectives and failure to produce adequateresources to support on-going efforts	Lacking integration withlawmakers (congress).	Open public meetings of regionalplanning bodies. Consultation atnational level. Strong stakeholderlobbying

Horizontally, all three processes are similarly well-integrated, but it has been achieved through different governance approaches. In Belgium a “Minister for the North Sea” was appointed to lead the process, Norway set one ministry (Environment) in charge to lead a multi-ministerial steering group in which the existing ministries were tasked to cooperate, while the US set down an Executive (Ocean Policy) Task Force, directly reporting to the Council on Environmental Quality in the White House and operating through government agencies on a (eco)regional basis. Both the Belgian and Norwegian processes are integrated on a national level, while the US proposed to integrate on a regional level (which is sensible as US regions are much larger than most European states). Horizontal integration across sectors relies strongly on each sector perceiving it to be treated in a fair and equitable manner. Therefore, meeting places and forums should be neutral – amalgamating the Arthurian ‘round table’ of Camelot that created peace among the noble but imperious knights closest to the king (in our examples the relevant ministers or president).

Vertically, Belgium and Norway are well-integrated from parliament (through the executive government levels) to stakeholders, while the US is well integrated from the Executive level downwards, but lacks integration (acceptance) from the law-makers (Congress).

Next to informal consultations with sectoral stakeholder groups, stakeholders also have a formalized role in some planning forums in Belgium. In Norway and the US, stakeholder participation remains informal and voluntary or self-selected (through consultations), although in the US stakeholder involvement is more comprehensive, reflected in the strong lobbying work of stakeholder groups to the government and the ability to file lawsuits based on administrative procedures and environmental compliance. Planning meetings are open to the public in the US, in contrast to Norway where meetings in governmental steering groups are held behind closed doors. The latter was also true for Belgium in the first MSP (2003–2005), but changed with the adoption process of the second MSP (2013–2014) when stakeholder participation and public consultation were included in the process. Although the current political situations may be regarded as challenging, the three cases illustrate the benefit of having a wide political backing to efficiently implement broad management processes like MSP. Where the MSP process tends to be top-down, formally defined processes and leadership are deemed necessary, while also bottom-up processes are highly valuable when it comes to integrating across vertical and horizontal scales.

In Belgium and Norway the process has led to effective and practical implementation of MSP, while in the US implementation is still pending. Our analysis shows that all three planning efforts can be regarded as well-integrated in intent, and that integration is important for a successful MSP. In addition to seeking and supporting political leadership, planners should keep in mind that processes and structures that facilitate effective integration are needed. Transparency and openness of access to stakeholders to make their perspectives be heard and analysed in the total process is a large part of what is required to ensure fair and equitable treatment, in relation to both horizontal and vertical integration.
